# Clinical, dermoscopic, and ultrasonic monitoring of the response to biologic treatment in patients with moderate-to-severe plaque psoriasis

**DOI:** 10.3389/fmed.2023.1162873

**Published:** 2023-06-26

**Authors:** Juncheng Wang, Qingli Zhu, Feng Li, Mengsu Xiao, Jie Liu

**Affiliations:** ^1^Department of Dermatology, State Key Laboratory of Complex Severe and Rare Diseases, Peking Union Medical College Hospital, Chinese Academy of Medical Science and Peking Union Medical College, National Clinical Research Center for Dermatologic and Immunologic Diseases, Beijing, China; ^2^Department of Ultrasound, Peking Union Medical College Hospital, Chinese Academy of Medical Sciences and Peking Union Medical College, Beijing, China

**Keywords:** psoriasis, biologics, dermoscopy, high-frequency ultrasound, secukinumab, correlation

## Abstract

**Background:**

Assessment of therapeutic response of psoriasis has relied traditionally on clinical observation, and effective non-invasive tools are desirable.

**Objectives:**

To investigate the value of dermoscopy and high-frequency ultrasound (HFUS) in the monitoring of psoriatic lesions treated with biologics.

**Methods:**

Patients with moderate-to-severe plaque psoriasis treated with biologics were evaluated by clinical, dermoscopic, and ultrasonic scores at weeks 0, 4, 8, and 12. Clinical scores, including Psoriasis Area Severity Index (PASI) and target lesion score (TLS), were evaluated at representative lesions. Dermoscopy was performed to assess the red background, vessels, and scales on a 4-point scale as well as the presence of hyperpigmentation, hemorrhagic spots, and linear vessels. HFUS was performed to measure the thicknesses of the superficial hyperechoic band and subepidermal hypoechoic band (SLEB). The correlation between clinical, dermoscopic, and ultrasonic evaluation was also analyzed.

**Results:**

In total, 24 patients were analyzed and achieved 85.3 and 87.5% reduction of PASI and TLS, respectively, after 12 weeks of treatment. The red background, vessels, and scales scores under dermoscopy were reduced by 78.5, 84.1, and 86.5%, respectively. Some patients developed hyperpigmentation and linear vessels after treatment. Hemorrhagic dots slowly subside over the therapeutic course. Ultrasonic scores were significantly improved with an average reduction of 53.9% in superficial hyperechoic band thickness and 89.9% in SLEB thickness. TLS in the clinical variables, scales in dermoscopic variables, and SLEB in ultrasonic variables decreased the most significantly in the early stage of treatment (week 4) with 55.4, 57.7, and 59.1% (*P* > 0.05), respectively. Most of the variables, including the red background, vessels, scales, and SLEB thickness, were strongly correlated with TLS. High correlations were also found between the SLEB thickness and the red background or vessels scores, and between the superficial hyperechoic band thickness and the scales scores.

**Conclusion:**

Both dermoscopy and HFUS were useful in the therapeutic monitoring of moderate-to-severe plaque psoriasis.

## 1. Introduction

Psoriasis is a chronic, recurrent, inflammatory skin disease that affects ~125 million people worldwide (1). The prevalence of psoriasis is ~3% in the United States (1, 2), 0.73–2.9% in Europe (3), and 0.12–1.49% in some provinces and cities in China (3). Plaque psoriasis is the most common variant of psoriasis and is characterized by erythematous scaly patches or plaques (1). Patients with psoriasis may show disfigurements and are at increased risk of comorbidities such as psoriatic arthritis, inflammatory bowel disease, metabolic syndrome, hypertension, and anxiety, all of which substantially diminish patients' quality of life (1).

To improve the lesions and quality of life of patients with psoriasis, in recent years, biologics, including TNF-α inhibitors (e.g., adalimumab), IL-17 inhibitors (e.g., secukinumab and ixekizumab), and IL12/23 inhibitors (e.g., ustekinumab), have been widely used. The efficacy and safety of biologics for the treatment of moderate-to-severe psoriasis are satisfactory (1), but problems with biologics are also encountered in clinical practice. For example, a small number of patients treated with biologics showed a poor treatment response and adverse effects and needed individualized maintenance therapy. In these patients, it is particularly important to timely and accurately follow their responses to treatments.

Traditionally, the assessment of therapeutic response has relied mostly on clinical observation, for example, through the use of the Psoriasis Area Severity Index (PASI) and Physician's Global Assessment (PGA) (1, 4). However, the above clinical assessments are subjective to a certain extent, and the observer's experience may lead to significant bias in the results. Therefore, through technological advances, novel and non-invasive tools, such as dermoscopy and high-frequency ultrasound (HFUS), have been developed to objectively monitor treatment response. In some studies, dermoscopy and HFUS have been shown to be promising tools for predicting and monitoring therapeutic outcomes (5–8).

Dermoscopy can be used to observe lesions from a view parallel to the skin surface and to show psoriatic hallmarks, including uniform dotted vessels, diffuse white scales, and a red background (5–7, 9). Hemorrhagic dots are also commonly seen and are often related to scratching (9, 10). Several studies have demonstrated that dermoscopic follow-up of psoriasis may facilitate treatment response assessment by showing changes in vessels or hemorrhagic dots (5, 7, 10).

HFUS can be used to observe lesions from a longitudinal view, and ultrasound characteristics of psoriasis include a superficial hyperechoic band and a subepidermal hypoechoic band (SLEB). Histologically, superficial hyperechoic band may correspond to thickened epidermis, mainly correlating with epidermal parakeratotic hyperkeratosis (7, 8). SLEB is related to acanthosis and downward elongation of rete ridges in the epidermis and inflammatory infiltration, telangiectasia, and edema in the papillary dermis. This intermingled structure results in a lower echo than the surrounding area. It is correlated with disease severity measured using the PASI and other scales (7, 8, 11, 12). In a previous study, HFUS evaluation showed a reduction in the thickness of the superficial hyperechoic band and SLEB in patients treated with ixekizumab (8).

As dermoscopy and ultrasound could detect subclinical changes in psoriasis, this study aimed to observe psoriatic lesions treated with biologics using dermoscopy and HFUS, to investigate the value of these tools in monitoring psoriasis treatment response, and to determine the correlation of observations made with these tools with clinical disease severity scores. In addition to standard parameters, we assessed additional parameters to monitor psoriasis.

## 2. Materials and methods

### 2.1. Patients and study design

For this prospective study, Asian patients (Fitzpatrick skin phototype III–IV) with moderate-to-severe chronic plaque psoriasis who were scheduled to receive a biologic were enrolled. The inclusion criteria were as follows: (1) patients who were aged at least 18 years and had moderate-to-severe plaque psoriasis; and (2) patients who had received no conventional systemic therapy for 4 weeks, biologic therapy for 6 months, or topical therapy for 4 weeks before treatment. The enrolled patients received standardized treatment with secukinumab (300 mg at weeks 0, 1, 2, 3, and 4, and then every 4 weeks), adalimumab (80 mg at week 0 followed by 40 mg every other week beginning at week 1), or ixekizumab (160 mg at week 0 and then 80 mg every 2 weeks). Dermatologists regularly assessed patients clinically and by dermoscopy and HFUS at weeks 0, 4, 8, and 12. The above observations were approved by the Medical Ethics Committee of Peking Union Medical College Hospital (JS-1233), and written informed consent was obtained from the patients.

### 2.2. Clinical evaluation

The PASI is a comprehensive evaluation of the severity of psoriatic plaques, including the intensity and extent of erythema, desquamation, and induration (13). A PASI or body surface area ≥3 is considered the criterion for moderate-to-severe psoriasis. For the patients in this study, two senior dermatologists evaluated the PASI at all time points and also the proportion of patients achieving an improvement in PASI of at least 75 or 90% at week 12 (PASI 75 or PASI 90). A representative lesion of each patient, which could be the most severe or thickest plaque, was then selected for the evaluation of the target lesion score (TLS). The TLS is an assessment of the severity of erythema, desquamation, and induration of local skin lesions on a 4-point scale, where 0 indicates none, 1 indicates mild, 2 indicates moderate, and 3 indicates severe, thus giving a total TLS score of 0–9 (7, 14). This representative lesion was also marked to ensure that the dermoscopy and HFUS measurements were obtained from the same location.

### 2.3. Dermoscopic and ultrasonic evaluation

**Figure 1:**
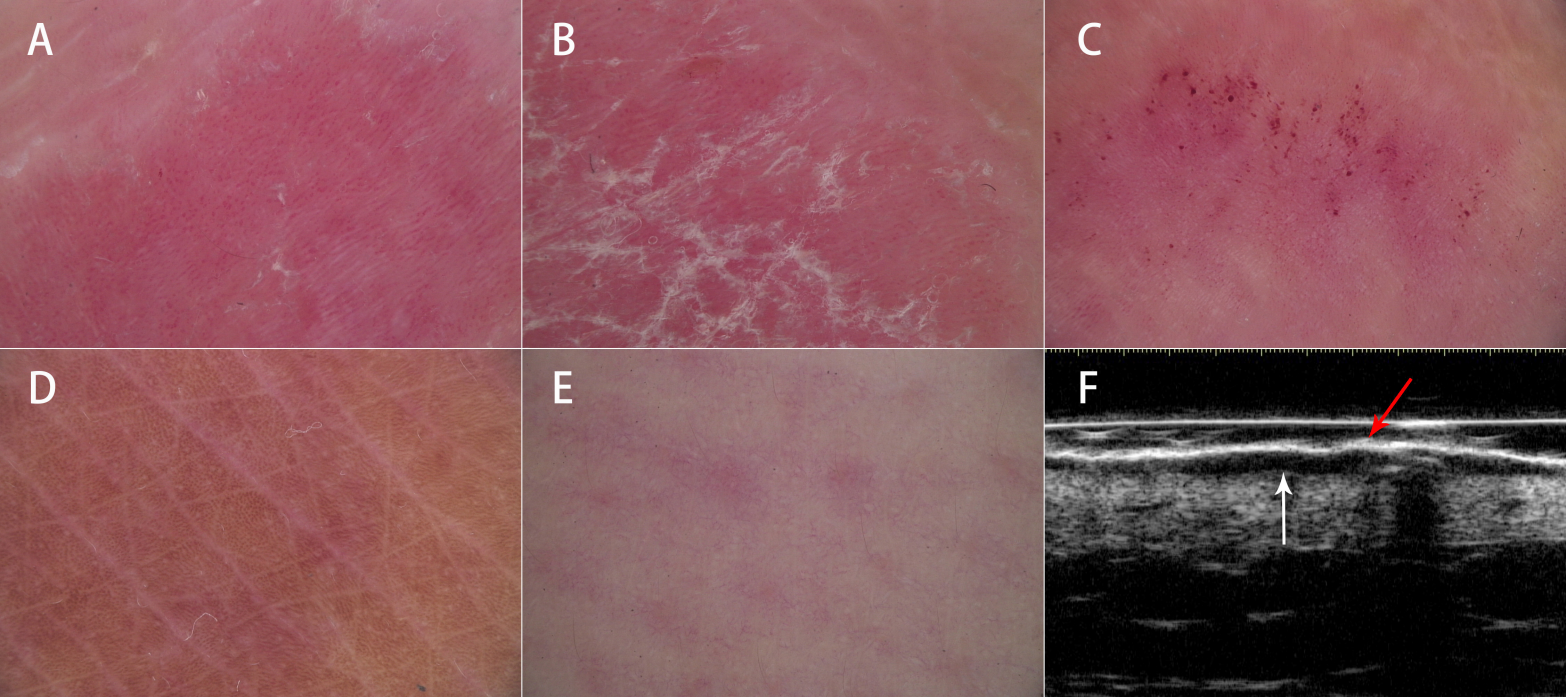
Dermoscopic and ultrasonic features of psoriasis. Dermoscopy showed **(A)** uniform dotted vessels on a red background, **(B)** diffuse white scales, **(C)** hemorrhagic dots, **(D)** hyperpigmentation, and **(E)** linear vessels. High-frequency ultrasound showed **(F)** a superficial hyperechoic band (red arrow) and a subepidermal hypoechoic band (white arrow).

The dermoscopic examination was performed using a digital dermoscopy system (MoleMax HD video dermatoscope, Digital Image Systems, Austria) in polarized light mode. All dermoscopic images were obtained by the same skilled operator to avoid interobserver variability. The two dermatologists then independently assessed each variable, namely, red background, vessels, and scales, on a 4-point scale (Figure 1). For example, the red background score ranges from 0 to 3 based on the color saturation of the red background, with 0 indicating no red background and 3 indicating bright red background; vessels of psoriatic lesions can be absent, few, clustered, or uniformly distributed according to their distribution and density, which were scored from 0 to 3, respectively. Target lesions were also evaluated for the presence or absence of hyperpigmentation, hemorrhagic spots, and linear vessels. In case of disagreements between the two dermatologists, a consensus reading was achieved through discussion.

The ultrasonic examination was carried out using an MD-300S type II diagnostic system (MEDA Co., Ltd., Tianjin, China) with 20 and 50 MHz probes. The thickness of the superficial hyperechoic band and SLEB were measured independently by two experienced doctors who had been trained in HFUS. A superficial hyperechoic band appears as an undulated or flat hyperechoic band on the surface of the lesion. A SLEB is an anechoic or hypoechoic band between the dermis and epidermis (8) (Figure 1). Ultrasonic measurements were obtained in triplicate, and the mean value was used. The final result for each lesion was the average of the measurements obtained by the two doctors.

### 2.4. Statistical analyses

Continuous data are expressed as the median, first quartile (Q1), third quartile (Q3), and range, and nominal data are expressed as numbers and percentages. Non-parametric tests (Wilcoxon) were conducted for comparisons between measurements obtained at different time points. Correlations among clinical, dermoscopic, and ultrasonic scores were calculated using Spearman's correlation coefficient, which was interpreted as follows: 0–0.4 indicated weak correlation, 0.4–0.6 indicated moderate correlation, 0.6–0.8 indicated strong correlation, and 0.8–1.0 indicated very strong correlation (15). A two-sided *P*-value of < 0.05 was considered statistically significant. All statistical analyses were performed using SPSS software (version 26.0; IBM Corp, Armonk, NY, USA).

## 3. Results

### 3.1. Patient characteristics

**Figure 2:**
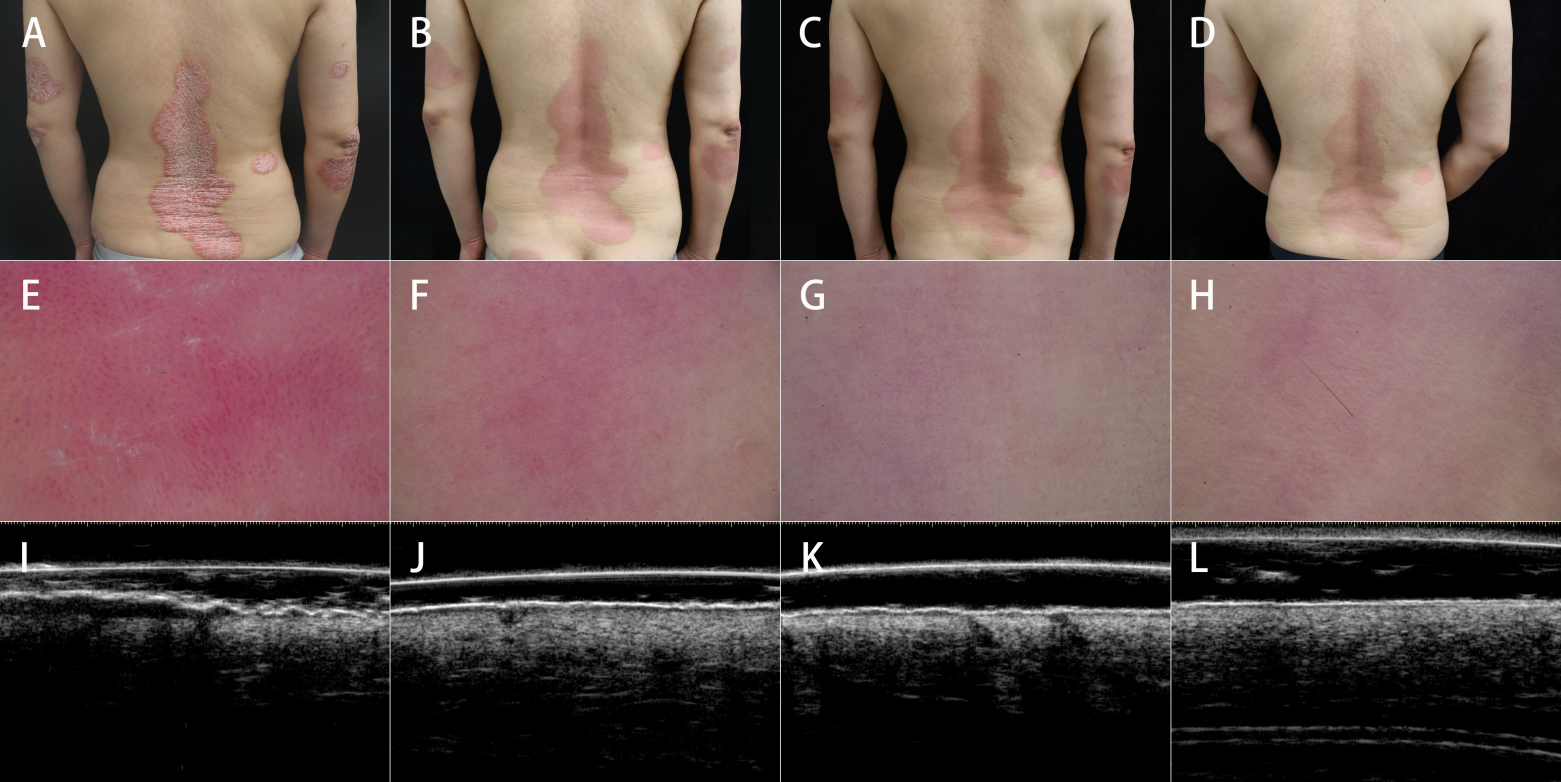
Clinical, dermoscopic, and ultrasonic assessments before and after treatment. Clinical assessment showed gradual improvements in erythema, scales, and induration from **(A)** baseline to **(B)** 4 weeks, **(C)** 8 weeks, and **(D)** 12 weeks after treatment. Dermoscopic assessment showed the normalization of the red background, vessel pattern, and scales and the appearance of linear vessels from **(E)** baseline to **(F)** 4 weeks, **(G)** 8 weeks, and **(H)** 12 weeks after treatment. HFUS showed significant improvements in the thicknesses of SLEB and superficial hyperechoic band from **(I)** baseline to **(J)** 4 weeks, **(K)** 8 weeks, and **(L)** 12 weeks after treatment, especially at the first 4 weeks of treatment.

A total of 27 patients aged 21–74 years meet the criteria and were enrolled, with 9 patients each receiving adalimumab, secukinumab, and ixekizumab as originally scheduled. Of the 27 patients, 3 patients treated with ixekizumab did not continue having completed week 8 due to job changes that caused them to leave this city, or living too far from the hospital for regular follow-up. Finally, 24 were included in the analysis (Table 1). As shown in Figure 2, patients underwent clinical, dermoscopic, and HFUS evaluations before and after treatment. Table 2 shows the scores of these evaluations at different time points.

**Table 1:** Demographic characteristics of the included patients.

**Characteristics**	***n* (%)**
Patients	24
**Treatment**	
Adalimumab	9 (37.5)
Secukinumab	9 (37.5)
Ixekizumab	6 (25.0)
**Sex**	
Male	16 (66.7)
Female	8 (33.3)
Age (years), *mean* ±*SD*	41.6 ± 15.7
**Location of the target lesion**	
Trunk	7 (29.2)
Upper limb	5 (20.8)
Lower limbs	12 (50.0)

**Table 2:** Clinical, dermoscopic, and ultrasonic evaluation scores at baseline and weeks 4, 8, and 12.

	**Baseline median (Q1; Q3) [range]**	**Week 4 median (Q1; Q3) [range]**	**Week 8 median (Q1; Q3) [range]**	**Week 12 median (Q1; Q3) [range]**
**Clinical score**				
PASI	19.8 (14.2; 30.9) [5.4–45.4]	8.9 (4.2; 16.8) [1.2–24.0]	3.2 (2.2; 9.6) [0.6–16.2]	1.8 (1.0; 4.2) [0.4–13.2]
TLS	7.0 (6.0; 8.0) [4.0–9.0]	3.0 (2.0; 4.0) [1.0–7.0]	1.0 (1.0; 2.0) [0–5.0]	1.0 (0; 1.0) [0–4.0]
**Dermoscopic score**				
Red background (0–3)	3.0 (2.0; 3.0) [2.0–3.0]	1.0 (1.0; 2.0) [0–3.0]	1.0 (1.0; 1.0) [0–2.0]	1.0 (0; 1.0) [0–1.0]
Vessels (0–3)	3.0 (3.0; 3,0) [2.0–3.0]	1.5 (1.0; 2.0) [0–3.0]	1.0 (0; 1.0) [0–2.0]	0 (0; 1.0) [0–2.0]
Scales (0–3)	2.0 (1.25; 3.0) [1.0–3.0]	1.0 (0; 1.0) [0–3.0]	0 (0; 1.0) [0–3.0]	0 (0; 0) [0–3.0]
Hyperpigmentation, *n* (%)	0	9 (37.5)	10 (41.7)	15 (62.5)
Hemorrhagic dots, *n* (%)	19 (79.2)	8 (33.3)	6 (25.0)	4 (16.7)
Linear vessels, *n* (%)	0	6 (25.0)	8 (33.3)	9 (37.5)
**Ultrasonic score**				
Thickness of superficial hyperechoic band (mm)	0.29 (0.24; 0.34) [0.16–0.50]	0.16 (0.14; 0.22) [0.10–0.32]	0.13 (0.12; 0.20) [0.08–0.26]	0.12 (0.11; 0.16) [0.08–0.20]
Thickness of SLEB (mm)	0.50 (0.39; 0.61) [0.16–1.36]	0.15 (0.11; 0.31) [0.06–0.76]	0.08 (0.06; 0.12) [0–0.50]	0.02 (0; 0.08) [0–0.32]

PASI, Psoriasis Area Severity Index; TLS, target lesion score; SLEB, subepidermal hypoechoic band.

### 3.2. Clinical monitoring during treatment

**Figure 3:**
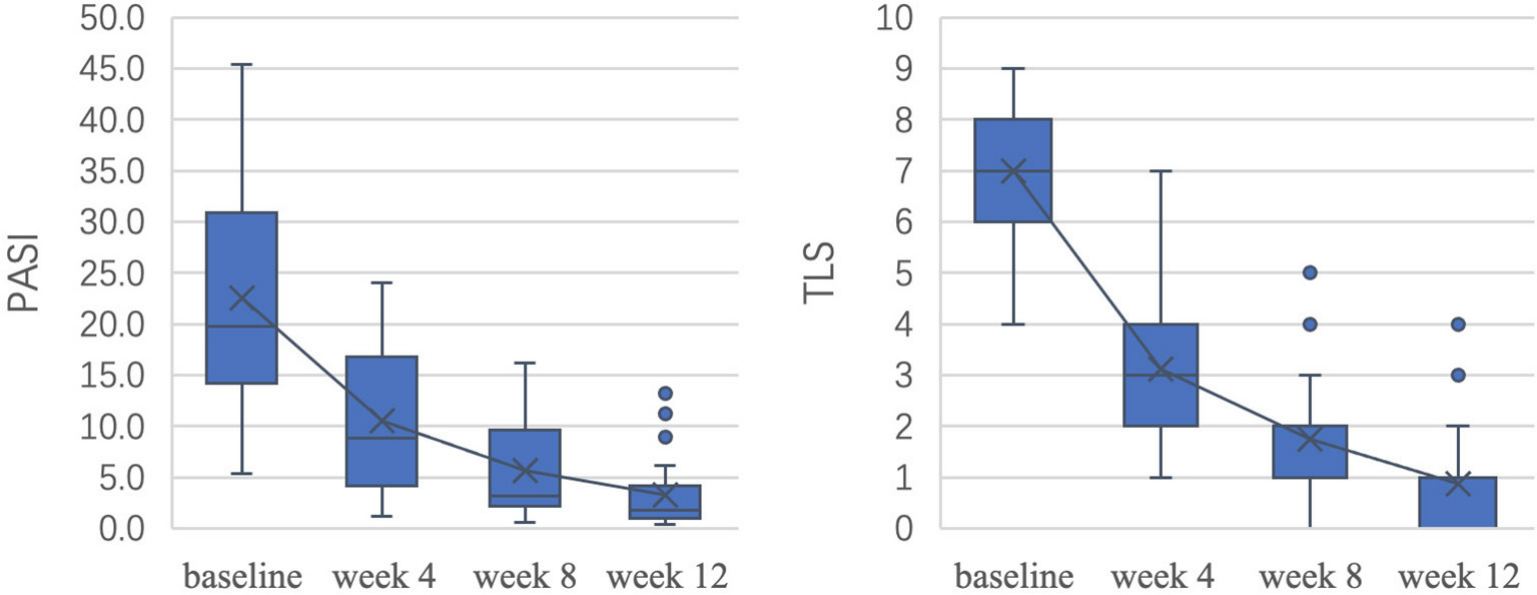
Reduction in clinical scores over the course of treatment.

Figure 3 shows the decrease in patient clinical scores throughout the course of treatment. After 12 weeks of treatment, the average reduction in the PASI was 85.4% (*P* < 0.05), 75.0% (18/24) of patients achieved a PASI 75 response, and 65.0% (13/24) of patients achieved a PASI 90 response. The TLS improved by 87.5% on average (*P* < 0.05). There was no significant difference between PASI improvement and TLS improvement (*P* > 0.05). The differences in PASI and TLS between adjacent time points were all statistically significant (*P* < 0.05).

### 3.3. Dermoscopic and ultrasonic monitoring during treatment

The dermoscopic variables showed significant improvement as well (Table 2). After 12 weeks of treatment, the red background score decreased by 78.5% (*P* < 0.05), the vessel score decreased by 84.1% (*P* < 0.05), and the scale score decreased by 86.5% (*P* < 0.05). Except for those with the scale scores between weeks 8 and 12, the *P-*values for the differences in the aforementioned variables between adjacent time points were <0.05.

**Figure 4:**
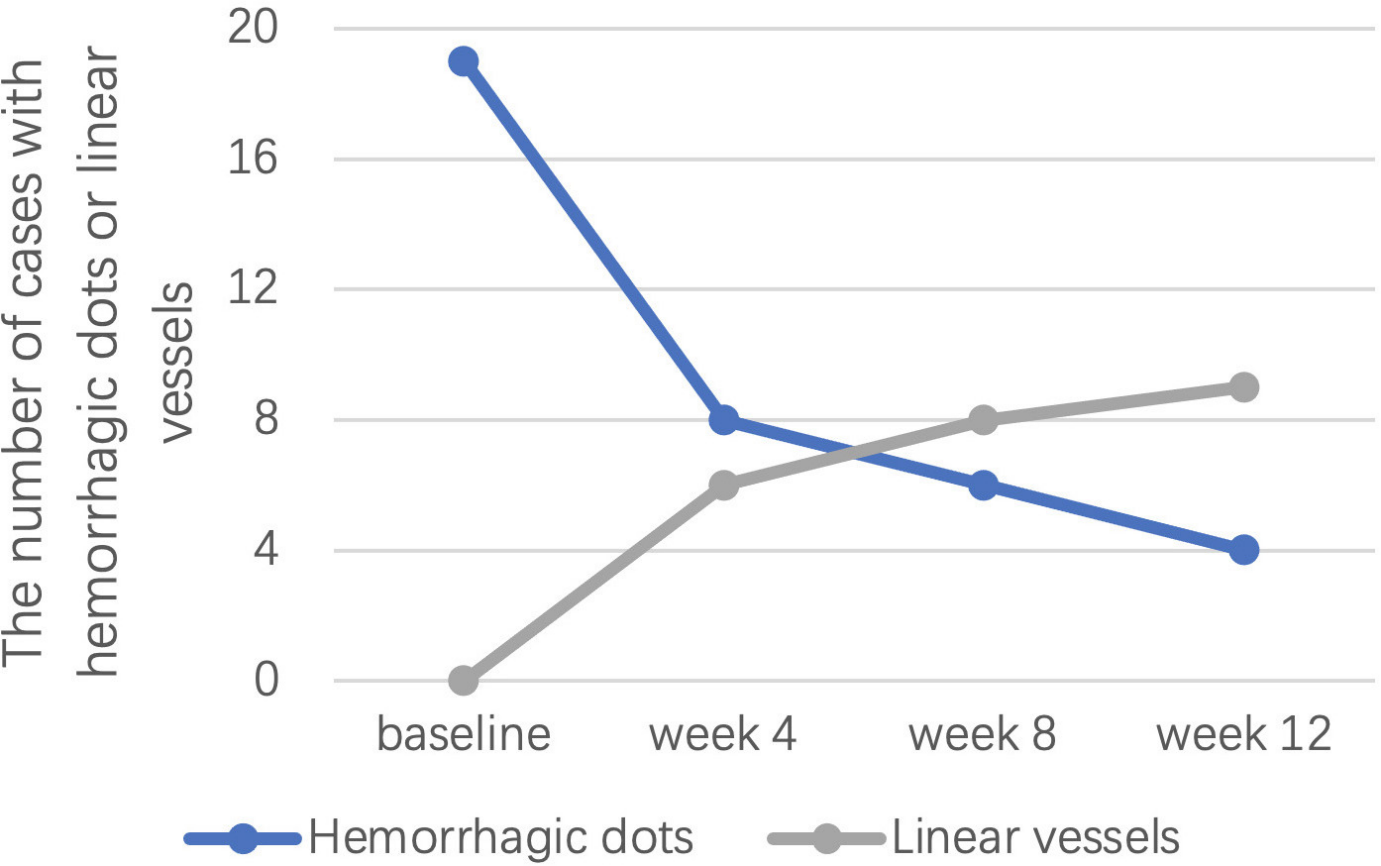
The number of cases with hemorrhagic dots or linear vessels at weeks 0, 4, 8, and 12.

Of note, hyperpigmentation was not observed for any patient at baseline, but 9 (37.5%), 10 (41.7%), and 15 (62.5%) patients developed hyperpigmentation after 4, 8, and 12 weeks of treatment, respectively. Linear vessels were also not observed for any patient at baseline but were observed in 6 (25.0%), 8 (33.3%), and 9 (37.5%) patients at weeks 4, 8, and 12, respectively (Figure 4). Hemorrhagic dots were observed in 19 (79.2%), 8 (33.3%), 6 (25.0%), and 4 (16.7%) patients at weeks 0, 4, 8, and 12, respectively (Figure 4).

**Figure 5:**
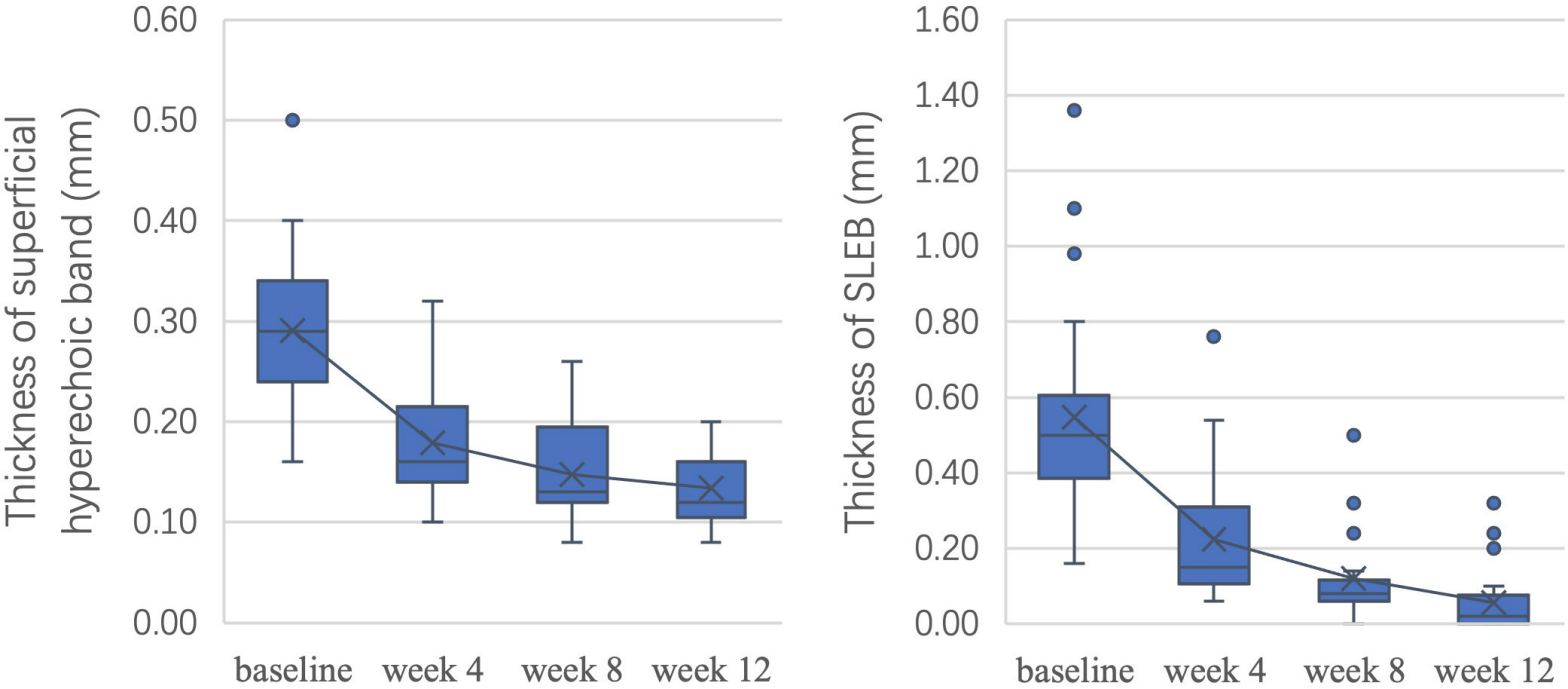
Ultrasonic score changes after treatment.

As shown in Figure 5, after 12 weeks of treatment, ultrasonic scores were significantly improved, with an overall reduction of 53.9% in the thickness of the superficial hyperechoic band (*P* < 0.05) and 89.9% in SLEB thickness (*P* < 0.05). All *P*-values were < 0.05 between adjacent time points.

**Figure 6:**
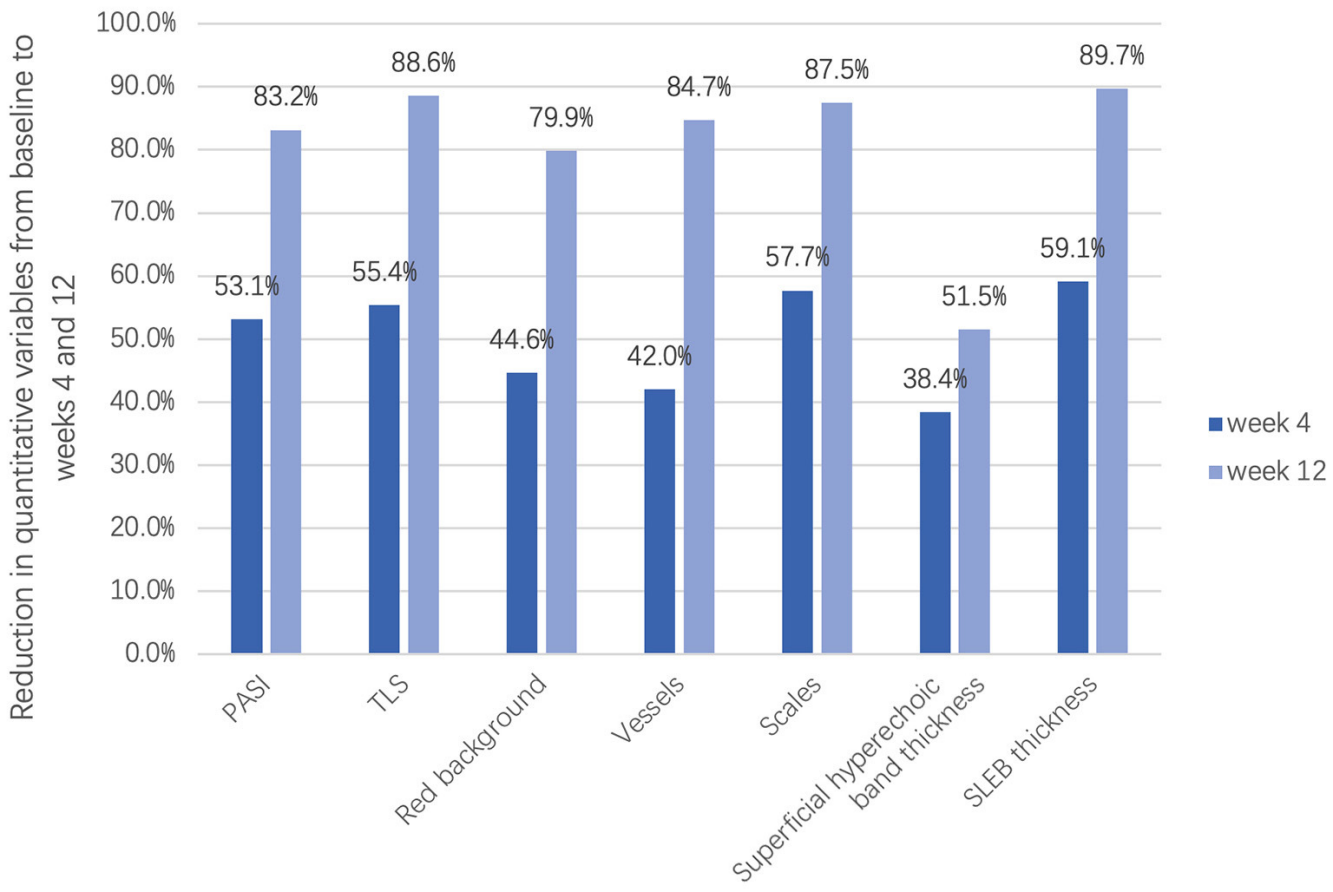
Reduction in clinical, dermoscopic, and ultrasonic quantitative variables in the early stage of treatment (week 4) and the late stage of treatment (week 12).

To measure the changes in skin lesions in the early stage of treatment, we calculated the changes in clinical, dermoscopic, and ultrasonic quantitative variables from baseline to week 4 (Figure 6). It was found that the TLS in clinical variables, scale score in dermoscopic vxariables, and SLEB in ultrasonic variables significantly decreased the most (55.4, 57.7, and 59.1%, respectively); however, there was no significant difference among them (*P* > 0.05). For dermoscopic variables, there was a significant difference between the improvement in the scale score (57.7%) and the improvement in either the red background score (44.6%) or the vessel score (42.0%) (*P* < 0.05). For HFUS variables, there was a significant difference between the improvement in SLEB thickness (59.1%) and that of superficial hyperechoic band thickness (38.4%) (*P* < 0.05).

### 3.4. Correlation between dermoscopic and HFUS variables and TLSs

To confirm the correlations between different evaluation variables, we calculated the correlation between TLSs and corresponding dermoscopic variables (red background, vessel, scale, and hemorrhagic dot scores) and ultrasonic variables (superficial hyperechoic band and SLEB thicknesses) at different time points (Table 3). There was no significant correlation between the TLS and the superficial hyperechoic band thickness at week 12 or the hemorrhagic dot score at baseline. Most of the variables, including the red background, vessel, and scale scores and SLEB thickness before and after treatment, were strongly correlated with the TLS. In addition, the TLS was very strongly correlated with the vessel score at all time points during treatment.

**Table 3:** Correlation of dermoscopic and ultrasonic variables with TLSs at baseline and posttreatment.

**Variables**	**Dermoscopic variable scores**				**Ultrasonic variables**	
	**Red background**	**Vessels**	**Scales**	**Hemorrhagic dots**	**Superficial hyperechoic band thickness**	**SLEB thickness**
TLS at baseline	0.647	0.412	0.614	0.267^*^	0.587	0.692
TLS at week 4	0.816	0.833	0.747	0.501	0.568	0.659
TLS at week 8	0.726	0.807	0.727	0.760	0.617	0.853
TLS at week 12	0.728	0.803	0.705	0.613	0.329^*^	0.661

TLS, target lesion score; SLEB, subepidermal hypoechoic band.

^*^P > 0.05 for this result.

In addition, we calculated the correlation between some dermoscopic and ultrasonic variables (Table 4). The correlation between the SLEB thickness and the red background or vessel score was moderate or above at all times. The correlation between the superficial hyperechoic band thickness in HFUS and the scale score in dermoscopy was moderate or above as well.

**Table 4:** Correlation between dermoscopic and ultrasonic variables at baseline and posttreatment.

**Variables at different time points**		**Red background**	**Vessels**	**Scales**
SLEB thickness	Baseline	0.524	0.420	-
	Week 4	0.520	0.464	-
	Week 8	0.724	0.678	-
	Week 12	0.411	0.759	-
Superficial hyperechoic band thickness	Baseline	-	-	0.508
	Week 4	-	-	0.496
	Week 8	-	-	0.669
	Week 12	-	-	0.522

SLEB, subepidermal hypoechoic band.

## 4. Discussion

Previous clinical scoring methods mainly evaluate the changes in psoriatic lesions from a macroscopic perspective but may ignore the subtle changes in local skin lesions, which may be closely related to prognosis, treatment adjustment, or recurrence prevention in psoriasis. Effective non-invasive techniques are useful in monitoring the alterations at a subclinical level and improving the management of psoriasis. In this study, we evaluated psoriatic patients treated with biologics using clinical evaluations, dermoscopy, and HFUS, which obtained good therapeutic monitoring results.

Dermoscopic variables have good and reliable value in monitoring during the treatment of psoriasis. The psoriatic lesions of all patients improved significantly after biologic treatment, and the red background, vessels, and scales also significantly improved under dermoscopy. Most of the dermoscopic variable scores were well correlated with the clinical scores at baseline and after treatment, which suggested that the dermoscopic manifestation score could well represent the actual condition of local lesions. The red background may be related to the expansion of dermal vessels and increased blood flow caused by the inflammation of psoriatic lesions, while dotted vessels histologically correspond to the tips of vertically arranged, dilated vessels in dermal papillae (16). Therefore, both a red background and vessels are closely related to the severity of skin lesions. Similarly, scales under dermoscopy are closely related to the parakeratosis and hyperkeratosis of the epidermis (16). In addition, scales were a more sensitive indicator of treatment response than a red background or vessels at the early stage with the highest improvement of 57.7% in dermoscopic variables (*P* < 0.05). These findings indicate that dermoscopy can well reflect the current condition of skin lesions for the monitoring of treatment response in psoriatic patients (5).

Compared to other dermoscopic variables, hyperpigmentation is a sign that is easily overlooked during dermoscopic monitoring and has not been reported in previous studies. We have found hyperpigmentation of lesions in some patients after biologic treatment, and there were individual differences in hyperpigmentation (17). However, postinflammatory hyperpigmentation is a reactive process resulting from increased melanin or abnormal distribution of melanin and is secondary to inflammatory cutaneous diseases, including psoriasis (18). Hyperpigmentation may also serve as an auxiliary indicator of treatment effectiveness, as when hyperpigmentation began to be observed under dermoscopy, other findings suggested that the lesion was gradually responding to treatment.

Similarly, linear vessels were also not a commonly observed dermoscopic feature in the lesions of psoriasis patients. In a previous study, the appearance of linear vessels suggested skin atrophy caused by potent topical steroids (19). However, biologic treatment did not induce the atrophy of the epidermis or dermis. Therefore, we speculated that although scales were reduced and the epidermis became thinner after treatment, the blood flow in the upper dermis did not fully normalize, which resulted in the horizontal vessels remaining dilated and linear vessels appearing under dermoscopy. In some patients, the linear vessels disappeared at the later stage of the 12-week follow-up. The appearance of linear vessels may be one of the indicators of an effective response to biologics and suggested that the current treatment should be maintained.

Hemorrhagic dots were an interesting dermoscopic variable in this study and presented not the same significance as that found in previous studies (6, 10). Lallas et al. suggested that hemorrhagic dots appearing in the early stage of treatment with biologics are associated with a good outcome (10). In this study, hemorrhagic dots were found in most patients at baseline and gradually decreased with the progression of treatment. Hemorrhagic dots may be related to scratching, dilated dermal papillary capillaries, and the thinning of the suprapapillary dermal plates, which could be improved by treatment. Therefore, we suggest that although not perfectly parallel to disease severity, hemorrhagic dots were more suitable as a variable for monitoring than for predicting treatment response in the Chinese population.

HFUS was a useful tool in monitoring treatment response. SLEB thickness under HFUS has often been used to monitor treatment response in psoriasis patients in previous studies (8). In this study, a moderate correlation was also observed between the TLS and SLEB thickness. SLEB thickness may be a good variable for assessing the severity of psoriatic lesions, but it is notable that a SLEB can also be present in other diseases, such as atopic dermatitis and skin photoaging (20). Although the thickness of the superficial hyperechoic band measured by HFUS was moderately correlated with the severity of lesions at baseline and in the early stage of treatment, there was no correlation at week 12. This finding may highlight that the scales regressed and the epidermal thickness normalized after 8–12 weeks of treatment. Therefore, the epidermal thickness of psoriatic lesions may enter a stationary phase, with 8 weeks of biologic treatment as the boundary. There was a correlation before that boundary but not after. Although the findings of some studies have suggested that HFUS may overestimate epidermal thickness in healthy humans, we believe that HFUS still has good value in assessing scales and a thickened epidermis (21). We also found that the dermal echo intensity of psoriatic lesions was lower than normal at baseline but increased slowly with treatment until it normalized. The lower dermal echo intensity was related to the infiltration of inflammatory cells and dermal edema, which increased the space between collagen fibers and decreased skin consistency. Biologic treatment can improve the above conditions and normalize the dermal echo density.

Previous studies have found that skin thickness measured by HFUS was the first parameter to improve compared with dermoscopic or clinical parameters with higher improvement (7). In this study, although the improvement in SLEB thickness was the greatest among the ultrasonic variables in the early stage of treatment, there was no significant difference between SLEB thickness and clinical and dermoscopic representative indicators, which may be related to the small number of patients and later first observation time. In short, SLEB and superficial hyperechoic band under HFUS can be used as variables for monitoring treatment effectiveness, and SLEB thickness is more sensitive in the early stage of treatment. In addition, the normalization of dermal echo density can also assist in observing treatment effectiveness.

To the best of our knowledge, this is the first study to explore the correlation between dermoscopic and ultrasonic parameters and to establish a connection between the findings of the two imaging techniques in psoriasis. The above results demonstrated that SLEB thickness was moderately or more correlated with red background and vessel scores. The correlation between superficial hyperechoic band thickness and the scale score was moderate for most patients throughout treatment. Although the observation perspectives of dermoscopy and ultrasound are different, the parameters of the two have some commonalities due to their similar histopathological backgrounds.

Although previous studies have used the above imaging methods for evaluation (6–8), few have used as many variables as this study to monitor changes in lesions. Compared with visual observation, the red background, vessels, scales, and hemorrhagic dots observed under dermoscopy help monitor the treatment response of psoriatic lesions, especially for local serious and refractory lesions. The appearance of linear vessels and hyperpigmentation are also helpful for the management of the treatment course. The thickness of SLEB and superficial hyperechoic band under HFUS were more quantitative and objective than naked-eye assessment and can detect deep changes in lesions. These non-invasive imaging technologies can serve as an important supplement to traditional visual observation, providing additional insights into the early detection and quantitative assessment of therapeutic effects from different perspectives. Therefore, the application of dermoscopy and HFUS in psoriasis can assist dermatologists in constructing a stereoscopic observation mode for the biologic treatment of moderate-to-severe plaque psoriasis.

This study has certain limitations. First, we included a small number of patients, which may affect the accuracy of the results. Second, to minimize drug interference, we included patients treated with only three representative biologics, which may in turn affect the results. Studies with larger sample sizes are yet to be conducted to confirm the results of this study.

In conclusion, this study identified the usefulness of both dermoscopy and HFUS in the therapeutic monitoring of patients with moderate-to-severe plaque psoriasis and the correlation among clinical, dermoscopic, and ultrasonic variables. These non-invasive modalities provide a new perspective and direction for the monitoring of psoriatic lesions treated with biologics, which may also be helpful for residents and general practitioners.

## Data availability statement

The original contributions presented in the study are included in the article/supplementary material, further inquiries can be directed to the corresponding authors.

## Ethics statement

The studies involving human participants were reviewed and approved by the Medical Ethics Committee of Peking Union Medical College Hospital (JS-1233). The patients/participants provided their written informed consent to participate in this study. Written informed consent was obtained from the individual(s) for the publication of any potentially identifiable images or data included in this article.

## Author contributions

FL, JL, and QZ conceptualized and designed the study. JW and FL collected data, conducted the analyses, and wrote the first draft of the manuscript. MX, FL, and JL critically revised the manuscript. All authors approved the final manuscript as submitted and agree to be accountable for all aspects of the work.
